# Does origin always matter? Evaluating the influence of nonlocal seed provenances for ecological restoration purposes in a widespread and outcrossing plant species

**DOI:** 10.1002/ece3.1817

**Published:** 2015-11-17

**Authors:** Jutta Reiker, Benjamin Schulz, Volker Wissemann, Birgit Gemeinholzer

**Affiliations:** ^1^ Institute of BotanyJustus Liebig University GiessenHeinrich‐Buff‐Ring 38D‐35392GiessenGermany; ^2^ Institute of Landscape Ecology and Resource ManagementInterdisciplinary Research Centre (IFZ)Justus Liebig University GiessenHeinrich‐Buff‐Ring 26‐32D‐35393GiessenGermany

**Keywords:** *Daucus carota*, local‐is‐best theory, microsatellite markers, nonlocal genotypes, population genetics, restoration

## Abstract

For restoration purposes, nature conservation generally enforces the use of local seed material based on the “local‐is‐best” (LIB) approach. However, in some cases recommendations to refrain from this approach have been made. Here we test if a common widespread species with no obvious signs of local adaptation may be a candidate species for abandoning LIB during restoration. Using 10 microsatellite markers we compared population genetic patterns of the generalist species *Daucus carota* in indigenous and formerly restored sites (nonlocal seed provenances). Gene diversity overall ranged between *H*_e_ = 0.67 and 0.86 and showed no significant differences between the two groups. Hierarchical AMOVA and principal component analysis revealed very high genetic population admixture and negligible differentiation between indigenous and restored sites (*F*_CT_ = 0.002). Moreover, differentiation between groups was caused by only one outlier population, where inbreeding effects are presumed. We therefore conclude that the introduction of nonlocal seed provenances in the course of landscape restoration did not jeopardize regional species persistence by contributing to inbreeding or outbreeding depressions, or any measurable adverse population genetic effect. On the basis of these results, we see no obvious objections to the current practice to use the 10‐fold cheaper, nonlocal seed material of *D. carota* for restoration projects.

## Introduction

In landscape and roadside verge restoration projects the use of nonlocal seeds has been – and often still is – common practice, as prices for nonlocal seed mixtures can be up to 10‐fold lower than for local provenances, and often large quantities of indigenous genotypes are unavailable (Burton and Burton 2002; Kettenring et al. 2014). However, introgression and hybridization between nonlocal and indigenous provenances can alter population genetic compositions as nonlocal genotypes might function as effective drivers for invasions below the species level (Jones 2013). This can lead to the homogenization, coexistence, or extinction of the regional and/or nonlocal gene pools with effects on the genotypic or allelic richness (Hughes et al. 2008). As the effects of nonlocal genotypes on the indigenous flora are still not well understood, nature conservation strategies proclaim the preservation and maintenance of local genotypic identities (Jones 2013). In some regions the use of indigenous genotypes for restoration purposes even becomes mandatory, for example, throughout Germany from 2020 onward as part of the nature protection and landscape conservation act (BNatSchG §40‐1 2010).

The use of local genotypes is justified by the biodiversity conservations' main strategy to preserve a region's genetic legacy resulting from a history of natural selection in local environments (Reed and Frankham 2001; Sackville Hamilton 2001; Jones 2013), hence to preserve indigenous provenances, based on the “local‐is‐best” (LIB) assumptions. By adhering to the LIB approach it is assumed that indigenous provenances are superior to nonlocal material with regard to fitness estimations or trait analyses (e.g., in relation to size and biomass, Leimu and Fischer 2008; Hereford 2009) as a result of local adaptation processes (Linhart and Grant 1996; Kawecki and Ebert 2004; Hereford 2009; Johnson et al. 2010). Theory predicts local adaptation to be positively correlated to increased genetic variation within populations and divergence between populations (Hereford 2009). Generally, larger populations have higher chances to be well adapted to their native environment as allelic divergence might support the presence of advantageous alleles (Whitlock 2003) and prevent the fixation of deleterious alleles (Lande 1994; Lynch and Milligan 1994; Whitlock 2000). Gene flow can hamper local adaptation by homogenizing allele frequencies and limiting the response to selection within environments (Stanton et al. 1997; Hendry and Taylor 2004; Kettenring et al. 2014), whereas environmental heterogeneity fosters local adaptation (Becker et al. 2006; Hereford and Winn 2008).

Even if local adaptation is ubiquitous (McKay et al. 2001; Angert and Schemske 2005; Kettenring et al. 2014) the relative strength and scale of adaptation varies across species and sites, and several authors predict local adaptation to be even less common than presumed (Leimu and Fischer 2008; Hereford 2009; Kettenring et al. 2014). Thus, strict adherence to the LIB approach for restoration may not evidently be the best choice for biodiversity conservation (Kettenring et al. 2014). And indeed, recommendations to refrain from the LIB approach were made, if (1) highly altered restoration environments radically differ from surrounding ecosystems (Kettenring et al. 2014), (2) locally adapted source populations as a result of strong directional selections are genetically depleted (Rice and Emery 2003; Broadhurst et al. 2008), and (3) an increase in local diversity by genetic reticulation between indigenous and nonlocal genotypes via hybridization could be beneficial for populations to adapt to future environmental changes (Rice and Emery 2003; Verhoeven et al. 2010; Sgrò et al. 2011; Breed et al. 2013).

Here, we test an additional argument to refrain from the LIB approach that is in contrast to current nature conservation practice, namely the case of widely distributed, common, generalist species that do not feature obvious indications of local adaptation. Therefore, populations of the widespread and outcrossing plant species *Daucus carota* were analyzed to compare genetic patterns of indigenous populations to those from sites formerly restored with nonlocal seed provenances.

Road construction and maintenance departments provided information about dates of restoration and applied seed mixtures of formerly restored sites in Central Germany. However, except that seed mixtures comprised nonlocal and non‐German genotypes, nothing is known about their exact origin.

*Daucus carota* (Apiaceae) was chosen for this study due to (1) its wide use in seed mixtures for herbal reintroductions, (2) its native abundance in a broad range of habitats, and (3) its common presence in the investigation area. Typical habitats of *D. carota* are meadows, thickets, and areas along railroads and roadsides with some kind of disturbance, while the species is also common in extensively managed grasslands. The species' native distribution covers large parts of Europe, Eastern and Central Asia, and the Mediterranean region with up to 10 poorly defined subspecies (Hegi 1964). *Daucus carota* is a biennial species, which is obligate cross‐pollinated with limited adaptations to species‐specific pollinators (Hegi 1964). The seeds are adapted to epizoochoric dispersal by featuring bristly hairs that protrude from the ribbed seed surface (Hegi 1964; Rong et al. 2010).

Molecular population analyses in *D. carota* have been applied previously, for example, by using random amplified polymorphic markers, inter simple sequence repeats (ISSR), microsatellite markers, and amplified fragment length polymorphisms (AFLP) – mainly focusing on cultivars or germplasm variability (Vivek and Simon 1999; Yan et al. 2009; Maksylewicz and Baranski 2013). However, some investigations also incorporated or screened wild taxa (Shim and Jørgensen 2000; Bradeen et al. 2002; Rong et al. 2010; Cavagnaro et al. 2011). Cavagnaro et al. (2011) designed polymorphic and robust PCR‐based microsatellite markers for *D. carota*, mainly to facilitate their inclusion in different maps as anchoring points for SSR tagging of phenotypic traits. We here use some of their developed microsatellite markers to investigate genetic diversity, population structure, and gene flow within and among populations.

## Materials and Methods

### Study sites

**Figure 1:**
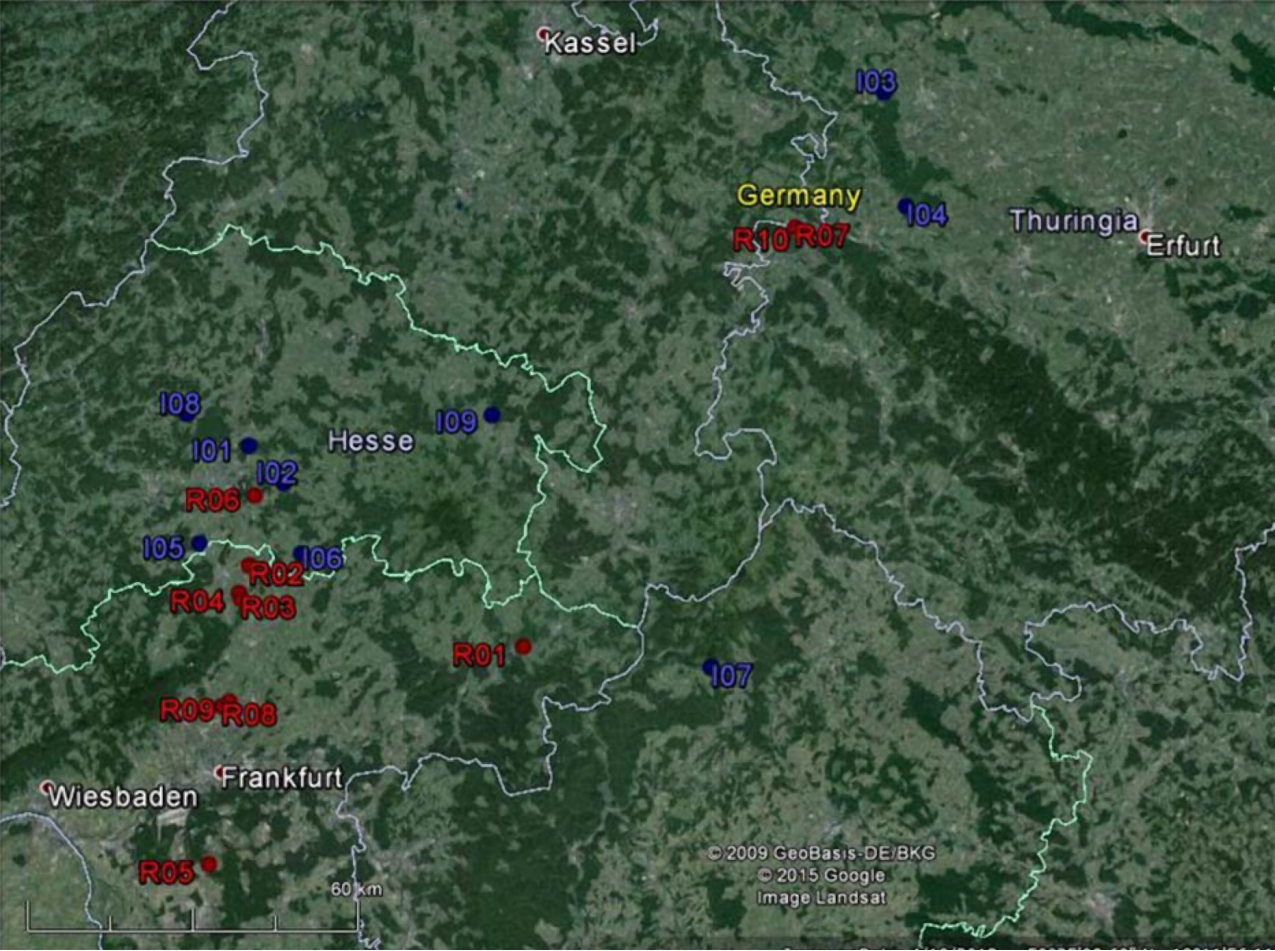
Map of sampled *Daucus carota* populations in the investigation area in Central Germany (Hesse, Thuringia, and Bavaria). Indigenous sites (*I*) are depicted in blue and restored sites (*R*) in red (see also Table 1). Source: Google Earth.

Road side authorities provided information about sites formerly restored with nonlocal seed material that comprised roadside verge restoration projects and compensatory sites. As roadside verges are chronically disturbed environments we decided to investigate compensatory sites only. Overall, we chose 10 restored sites (*R*) situated in Central Germany (Central and South Hesse, W‐Thuringia, NW Bavaria) within an investigation area of approximately 200 × 200 km^2^ in a comparatively sparsely populated hilly region. *R*‐sites cover an area between 0.5 and 2 ha and were restored between 1996 and 2004 (Table 1, Fig. 1). All sites were converted from arable land to compensatory sites and are adjacent either to woodlands, meadows, or agricultural fields, with corn, rapeseeds, and cereals being the most common crops in the region. Arable land in this region is commonly being plowed at least once a year. Biennial species which start flowering and fruit set in the second year normally do not survive plowing and thus have limited chances to substantially contribute to the soil seed bank. However, we cannot totally exclude indigenous *D. carota* seed dispersal from adjacent fields that may have contributed to the seed bank and recolonized once restored.

**Table 1:** Overview of surveyed *Daucus carota* populations

Population code	Location	Latitude	Longitude	Sample number	Date of restoration
I01	Daubringen	50.640255	8.739055	19	–
I02	Reiskirchen	50.581666	8.829360	18	–
I03	Eichsfeld	51.220721	10.358348	18	–
I04	Hainich	51.036522	10.415168	13	–
I05	Niederkleen	50.480773	8.616436	18	–
I06	Hungen	50.467687	8.877661	17	–
I07	Geroda	50.292924	9.920461	17	–
I08	Kirchvers	50.690361	8.579271	17	–
I09	Lauterbach	50.696151	9.359601	15	–
R01	Steinau	50.323347	9.446011	16	1994
R02	Griedel	50.447305	8.745246	17	1996
R03	Bad Nauheim	50.392279	8.726213	19	1996
R04	Bad Nauheim	50.402702	8.720849	18	1996
R05	Egelsbach	49.962194	8.655639	20	1998
R06	Fernwald	50.560872	8.755674	20	2003
R07	Herleshausen	51.002248	10.130403	17	2003
R08	Eschbach	50.218377	8.682396	16	2004
R09	Eschbach	50.226271	8.701537	18	2004
R10	Herleshausen	50.995496	10.153041	20	2004

*R*, Restored populations; *I*, indigenous populations.

As the main motivation for site selection was good documentation of site history, populations are not regularly spaced throughout the investigation area. For each site, information about the year of restoration as well as the presence and percentage of *D. carota* in the seed mixture are available (0.1% since 1988 FLL Bonn).

During data analysis the restored site R04 turned out to markedly differ from all others site. Hence, some calculations were additionally executed for a subset of *R*‐populations excluding the outlier R04.

For comparison, we investigated nine indigenous sites (*I*), as representatives of the local genotypic diversity: four meadows which are mostly maintained under the Habitats Directive of Natura 2000, and five protected nature reserve areas (Table 1). All *I* sites have not been modified or re‐sown during the last 60 years (Kunzmann et al. 2010). They were chosen due to their regional vicinity to the restored sites. Notwithstanding, a distance of at least 9 km between indigenous and restored sites was kept. In addition, a minimum distance of 200 m to any other adjacent population of cultivated relatives was taken into account to minimize potential effects of hybridization (Posselt 2000; Kunzmann et al. 2010). We are aware that potential hybridization between wild and cultivated carrots in the study region cannot be excluded. However, in a highly anthropogenic influenced, patch‐work structured landscape with mainly small isolated nature conservation areas, our approach seemed to be the best trade‐off to define “regional species diversity.”

On each site, leaf material of 20 individuals was sampled along transects and immediately dried in silica gel. Distances between sampled individuals within populations were at least 2 m to optimize coverage of site specific populations' genetic diversity.

### Molecular methods

Approximately 10 mg silica dried leaf material per individual was used for DNA extraction. The DNeasy plant Mini Kit (Qiagen, Hilden, Germany) was applied according to the manufacturer's instructions. DNA was diluted to 3–10 ng/*µ*L. Ten microsatellite primer combinations, developed by Cavagnaro et al. (2011) for its use on *D. carota*, were optimized to suite for this investigation (Table 2). The PCR mixture with a total volume of 20 *μ*L contained: 7.7 *μ*L dd H_2_O, 1 *μ*L HEX or FAM fluorescence‐labeled forward primer (5 pmol/*μ*L), 1 *μ*L reverse primer (5 pmol/*μ*L), 0.4 *μ*L BSA (10 ng/*μ*L), 4*μ*l Betain Monohydrat (5 mol/L), 2.4 *μ*L dNTPs (2 mmol/L), 0.5 *μ*L (5 U/*μ*L) DreamTaq polymerase (Fermentas, Cologne, Germany), 2 *μ*L 10 × DreamTaq PCR buffer (Fermentas), and 1 *μ*L of the diluted genomic DNA. PCR was conducted with an Eppendorf‐Gradient‐Mastercycler. The PCR program was 2 min at 95°C for initial denaturation, followed by 35 cycles of 30 sec at 95°C, 30 sec at the annealing temperature of each primer pair (54–57°C, Table 2), and 45 sec at 72°C; followed by a final extension at 72°C for 15 min. PCR products were sent to LGC Forensics (Cologne, Germany) for fragment visualization.

**Table 2:** Microsatellite marker comparison of this investigation (JR) and by Cavagnaro et al. (2011) (C), with locus name, microsatellite motifs (SSR motifs), annealing temperature, size range in base pairs (bp), number of alleles, Neis unbiased gene diversity (*H*_e_), and the number of null alleles and rare alleles found in this investigation in one sample (in 1), or only in two samples (in 2). Loci that significantly deviated from the Hardy–Weinberg (HDW) expectations are shown in bold

Locus	SSR motifs	Annealing temperature (°C)	Size range (bp)		Number of alleles		*H*_e_		Null alleles	Rare alleles		HDW		
			C	JR	C	JR	C	JR		In 1	In 2	*χ* ^2^	df	*P*
gssr3	(AG)6tgga(GGAG)3	54	285–335	266–322	20	25	0.86	0.66	16	6	1	5.004	10	0.891^ns^
gssr4	(TCTA)21	55	253–320	230–322	29	22	0.91	0.91	1	3	1	56.050	55	0.435^ns^
gssr6	(TC)9a(CT)11 (CAGTAG)4	50	283–331	262–312	19	21	0.89	0.80	13	1	0	**68.056**	**36**	**0.001** ***
gssr9	(TG)13ata(TATG)10gatgg(ATGT)3	54	281–337	259–327	22	31	0.77	0.91	3	1	3	**137.333**	**91**	**0.001** ***
gssr16	(TG)9tacgc(ATGT)3	57	229–346	207–285	16	34	0.82	0.85	4	3	3	51.477	66	0.905^ns^
gssr35	(GA)13	55	144–219	148–230	21	27	0.87	0.88	13	2	3	60.878	45	0.057^ns^
gssr65	(TG)8	57	404–433	372–419	10	12	0.79	0.74	6	1	0	20.969	21	0.461^ns^
gssr85	(TCTA)4tttatca(ATCT)4gtctgtcta(TCTG)3	54	219–294	196–316	14	25	0.84	0.90	1	1	0	40.500	45	0.663^ns^
gssr107	(ATAC)8(ACAT)4	54	no data	219–299	no data	15	no data	0.53	0	0	1	16.650	10	0.082^ns^
gssr111	(ATAC)3atccatc(CATA)9tat(CA)20	55	284–390	316–380	21	28	0.79	0.92	2	0	0	98.500	91	0.277^ns^

HDW correlation coefficient; *** very significant *P*<0.001; ns = no significant.

### Data analysis

Microsatellite data were processed with GeneMarker^®^ V1.90 (software SoftGenetica, LLC, State College, PA). The lengths of the DNA fragments were standardized using ROX 500. For evaluation, fragments were recorded in a codominant data matrix. Genetic diversity within populations was estimated as number of different alleles (*N*_a_), number of effective alleles (*N*_e_), Shannon's information index (*H′*), and observed (*H*_o_) and unbiased expected heterozygosity (*uH*_e_) using GenAlEx 6.5 (Peakall and Smouse 2012). Significance of differences between diversity estimates with small sample sizes was tested with two‐tailed *t*‐tests. Single sample *t*‐tests were applied and the nonparametric Mann–Whitney *U*‐test was used to analyze differences among groups (all http://www.socscistatistics.com/tests/mannwhitney/Default.aspx). Genetic variation among groups of indigenous and restored populations (*F*_CT_), among populations within groups (*F*_SC_) and within populations (*F*_ST_) was partitioned with hierarchical analysis of molecular variance (AMOVA) using ARLEQUIN 3.5.1.2 (Excoffier and Lischer 2010). Significance levels were determined after 9999 permutations. Furthermore, clustering of samples was visualized with principal component analysis (PCA) using the R package ADEGENET v1.4‐2 (Jombart 2008).

## Results

Data of 333 individuals from 19 different sites with 10 microsatellite markers each were analyzed. In total, 20 individuals per site were sampled; however, for some sites data retrieval could only be achieved for fewer specimens (Table 1, Fig. 1).

### Microsatellite statistics

Microsatellite statistics (Table 2) for allele size ranges of markers, number of alleles, and *H*_e_ values partly differed from earlier publications (Cavagnaro et al. 2011). However, most likely this is due to different surveyed wild genotypes as well as differences in the genotyping facility (i.e., equipment and software). A total of 257 alleles were generated from the 10 microsatellite markers (mean 24.0 ± 6.78 alleles per locus). Allele ranges had a mean of 67.0 ± 21.9 bp (SD). Total number of samples producing null alleles was 59 (22.96%) of the 257 alleles. Seven markers were null at more than one sample. The mean number of null alleles per locus was 5.9 ± 5.8 SD. Rare alleles made up 30 of the 257 total alleles (mean 3.0 ± 3.1 SD). Locus GSSR 6 and GSSR 9 deviated significantly from Hardy–Weinberg expectations. None of the applied microsatellite markers revealed fixation for different alleles in any of the screened populations.

### Population statistics

Overall, *H*_e_ values ranged from 0.67 to 0.86 (Table 3) with an average expected heterozygosity of *H*_e_ = 0.81. Thus, all sampled individuals revealed a high chance of being heterozygous. When only indigenous populations were considered, average expected heterozygosity increased slightly to *H*_e_ = 0.82, while for restored sites it was *H*_e_ = 0.80. Overall, *t*‐tests revealed no significant differences in diversity estimates between indigenous and restored populations (*P *<* *0.05). Highest number of effective alleles (*N*_e_) and highest *H*_e_ values were found in an indigenous population (I03: *H*_e_ = 0.89; *N*_e_ = 8.3). In contrast, highest number of different alleles (*N*_a_) was found in a restored population (R05; *N*_a_ = 12.4). Lowest *N*_e_, *I*,* H*_o_, and *H*_e_ were found in population R04. Single sample *t*‐tests revealed R04 to diverge significantly from all other populations (*N*_a,_ *N*_e,_ *H′, H*_o_, and *uH*_e_; *P* ≤ 0.01). However, the equality of variances between all indigenous and restored populations with or without R04 was not affected (*P* < 0.05).

**Table 3:** Measures of *Daucus carota* within‐population diversity

Population code	*N*_a_	*N*_e_	*H′*	*H*_o_	*uH*_e_
I01	10.2	5.7	1.9	0.77	0.83
I02	11.1	7.0	2.1	0.75	0.86
I03	11.9	8.3	2.2	0.75	0.89
I04	10.5	6.5	2.0	0.81	0.86
I05	11.2	7.7	2.2	0.77	0.88
I06	10.5	5.8	1.9	0.74	0.81
I07	8.1	5.4	1.8	0.79	0.81
I08	11.7	7.7	2.2	0.78	0.87
I09	9.7	5.9	1.9	0.79	0.81
R01	10.5	5.6	2.0	0.77	0.82
R02	9.2	5.1	1.8	0.74	0.80
R03	9.7	5.2	1.9	0.72	0.80
R04	6.6	3.5	1.4	0.61	0.69
R05	12.4	8.1	2.2	0.83	0.87
R06	11.0	6.8	2.0	0.74	0.85
R07	11.6	6.5	2.1	0.76	0.85
R08	11.1	6.5	2.0	0.76	0.84
R09	11.4	6.9	2.1	0.79	0.87
R10	11.4	5.6	2.0	0.74	0.81
Average all	10.5	6.3	1.99	0.76	0.83
Average *I*	10.5	6.7	2.02	0.77	0.85
Average *R*	10.5	6.0	1.95	0.75	0.82
Average *R*–Pop R04	10.9	6.2	2.01	0.76	0.84

*N*_a_, number of different alleles; *N*_e_, number of effective alleles; *H*, Shannon's information index; *H*, observed heterozygosity; *uHe*, unbiased expected heterozygosity; *R*, restored populations; *I*, indigenous populations.

Hierarchical AMOVA revealed that most genetic variation resided within populations (95.6%), whereas only 4.1% explained differences among populations within groups and only 0.2% among indigenous and restored populations (Table 4). Interestingly, when R04 was excluded from the dataset differences between indigenous and restored sites diminished (data not shown). Thus, indigenous and restored sites can more or less be considered as part of one single, random mating population with arbitrary groupings of subpopulations. At the individual group level, differentiation among indigenous populations (*F*_ST_ = 0.030, *P* < 0.01) was lower than among restored populations (*F*_ST_ = 0.055, *P* < 0.001). However, again excluding R04 from the dataset led to comparable values for restored sites (*F*_ST_ = 0.034, *P* < 0.01), substantiating the different population genetic pattern of R04 (Table 4).

**Table 4:** Summary of hierarchical AMOVA results for 19 *Daucus carota* populations grouped for indigenous (*n* = 9) and restored populations (*n* = 10)

Source	*V*	% Total	*P*	*F*_ST_ statistics
Among groups	0.010	0.2	0.049	*F*_CT_ = 0.002
Among populations within groups	0.174	4.1	<0.001	*F*_SC_ = 0.041
Within populations	4.023	95.6	<0.001	*F*_ST_ = 0.044

**Figure 2:**
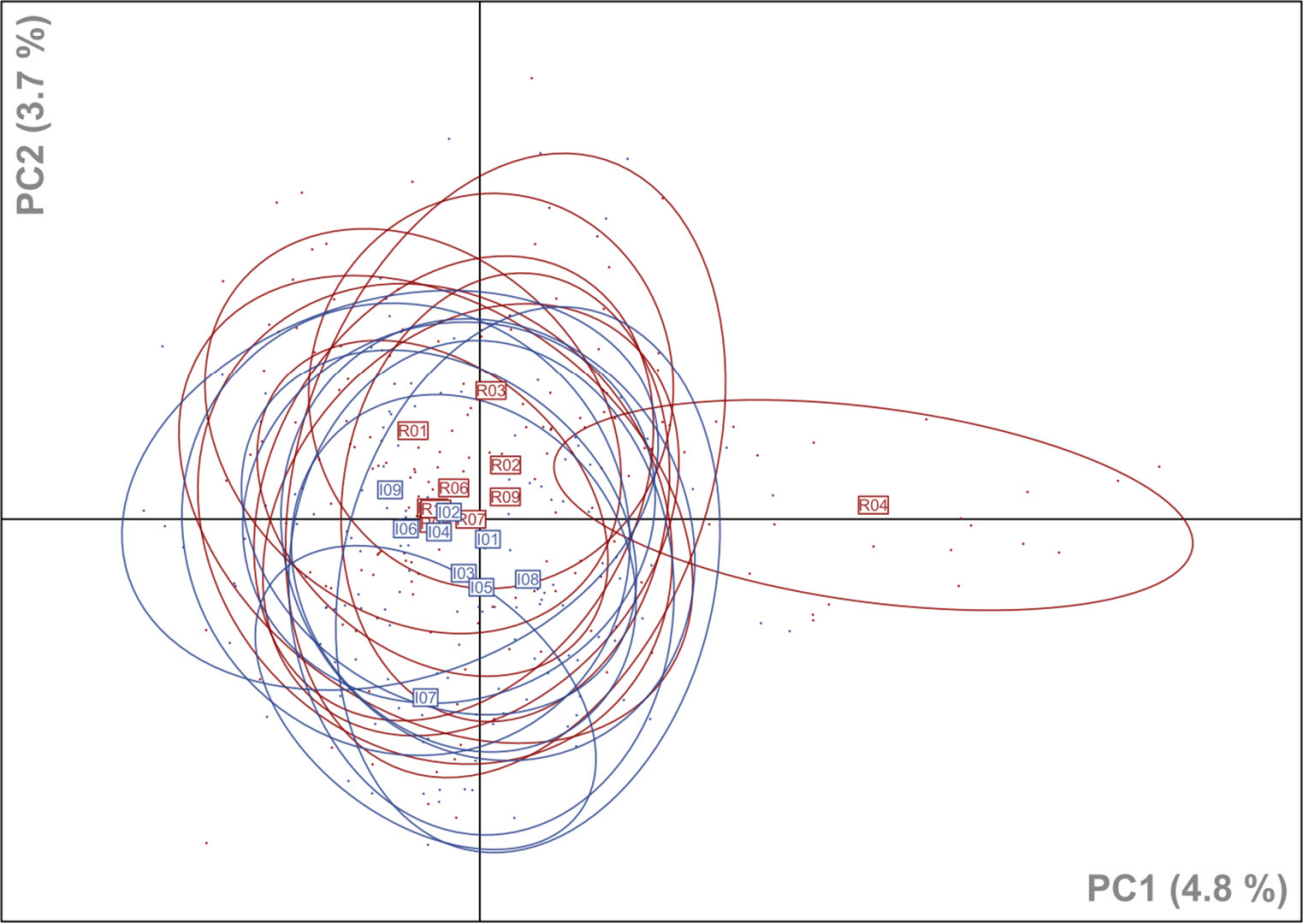
Principal component analysis (PCA) depicting the genetic structure in populations of *Daucus carota*. Indigenous populations (*I*) are indicated in blue and restored populations (*R*) in red. Label positions represent the centroids of the respective population. Inertia ellipses indicate dispersion of samples in relation to mean coordinates and include approximately three fourth (76%) of all individuals for each population.

AMOVA results were strongly corroborated by PCA analysis (Fig. 2). There was no distinct clustering of populations, and only centroids of indigenous and restored populations tended to be separated along the second component. One exception was again population R04. The first three components accounted for 4.8%, 3.7%, and 3.4% of genetic variation.

Genotypic population affiliation in general could not be explained by the year of restoration (see Table 1) or genetic diversity, with the exception of population R04.

## Discussion

Our study was intended to test if the biodiversity conservation strategy to preserve the region's genetic legacy by using local genotypes is justified for widely distributed, common and generalist plant species, like *D. carota*. We therefore compared population genetic patterns of indigenous populations with populations that were restored with nonlocal seed provenances between 1994 and 2004, hence after several years of establishment.

Throughout our whole investigation area we found very low population differentiation among the sampled groups of *D. carota* populations. Overall, population genetic patterns are indicative for high genetic admixture between indigenous as well as formerly restored sites with no clear evidences of local genetic adaptation. Bradeen et al. (2002) already postulated the population genetic pattern of wild carrot to be genetically nonstructured. This is partly supported by Rong et al. (2010) who detected weak but significant genetic structures in Dutch wild carrot populations. In contrast Shim and Jørgensen (2000) found pronounced genetic structures; however, this is an investigation in Denmark toward the species' northernmost distribution range where species‐specific parapatric local adaptations are more likely to occur (Brown and Amacher 1999; Sagarin and Gaines 2002; Eckert et al. 2008; Sexton et al. 2009).

Most of the molecular variation in our analysis occurs within populations, which is common for outcrossing species (Aavik et al. 2012). This is supported by Rong et al. (2010), who revealed distinctive long distance pollen dispersal of at least 4 km in *D. carota* and claimed that most offspring from a maternal plant resulted from different paternal individuals. They estimated outcrossing rates of 96% for wild carrot populations and explained this high outcrossing rate by the strongly proterandrous inflorescence with stigmas only becoming receptive when anthers of all stamens in the umbel have completed dehiscence (Koul et al. 1989). Thus, the specialized pollination mechanism triggers pollen‐mediated gene flow among distant individuals and weakens spatial genetic structures (Umehara et al. 2005; Rong et al. 2010).

Assumptions that the observed genetic patterns partly derived from local genotypes from the soil seed bank without successful propagation of foreign genotypes cannot totally be rejected, as *D. carota* seeds have the potential to survive several years under field conditions (Gross and Werner 1982; Thompson et al. 1993; Clark and Wilson 2003; Rawnsley et al. 2003). Widely practiced yearly plowing prior to restoration must have hampered *D. carota* persistence on the sites, but we cannot totally exclude indigenous seed dispersal from adjacent fields that may have contributed to the seed bank and recolonized prior or once restored.

The detected slight differences in diversity estimates between indigenous and restored populations could potentially pinpoint to novel genotype introductions in the region, however, from very similar population genetic origin. Umehara et al. (2005) already stated that carrots, even cultivated varieties and wild carrots, have extremely wide gene diversity (Rong et al. 2010). This is supported by our analysis which revealed substantial levels of genetic diversities on different scales, such as (1) within populations, (2) between individuals of the indigenous sites but also on the restored sites, and (3) throughout the investigation area. The overall detected genetic diversity in our analysis is in accordance to other investigations in wild carrot, for example, by Rong et al. (2010), and confirms earlier hypotheses. Markedly lower overall values were reported by Shim and Jørgensen (2000) and Bradeen et al. (2002), however, with quantitative molecular markers (AFLP, ISSR).

The highest allelic diversities were found in indigenous populations (I03, I05, and I08) and in one restored site (R05), which was established in 1998. As no information about the initial genotypic diversities of the nonlocal seed mixtures at the restored sites are available, it remains rather speculative if the current findings are the results of slightly lower initial genotypic diversities in the seed mixtures or are due to selection processes in the new habitats.

The lowest allelic diversities were found in one restored population R04, which diverged substantially in its population genetic pattern from all other investigated sites. Restoration of R04 took place in 1996 together with population R03, which is in close vicinity. The executing authority was in both cases the same. Thus, the detected difference in population genetic diversity is rather unlikely to originate from differences in the initial seed mixtures. The comparatively low values of within‐population diversity with the extremely low values of different alleles and effective alleles in R04 could be indicative for high inbreeding. R04, with approximately 1 ha, is nowadays predominantly covered in scrub vegetation (approximately 80%) and *D. carota* only remained as a remnant within grassland on the margins of the site, where other dicots are scarce, too. Due to intensively used agricultural fields in close vicinity, there are no other potential habitats for *D. carota* within a radius of 1 km. Even if we consider that pollinators do have longer flight ranges, site visitation for pollination might be comparatively rare.

We found no obvious signs that the introduction of nonlocal seed material in the course of restoration purposes contributed to the regional overall genetic diversity of the species. This finding is in contrast to current assumptions that “the diversity of the original source population is a critical consideration for restoration purposes, and that the starting pool of genetic diversity governs the performance of a reintroduced population for a long time” (Falk et al. 2001). However, it still remains unclear, if the initial genetic diversity in the nonlocal seed mixtures was indeed that different from indigenous populations. Gemeinholzer and Bachmann (2005) also conducted population genetic analyses in the comparable abundant, widespread, and generalist *Cichorium intybus* L., and discovered high genetic similarity in populations from Germany, Italy, Croatia, and Uzbekistan, even with increasing geographic distances. Thus, as population genetic diversity is the result of the accumulations of neutral substitutions or diversifying or frequency‐dependent selection, one might have to refer to “nonlocal” as a sweeping term, dependent on the degree of adaptation and selection in the respective target species, without narrowing it down to geographic vicinity, which currently is the common approach in many nature conservation strategies.

## Conclusion

For restoration practitioners the use of local seeds has become a common objective (e.g., Broadhurst et al. 2008; Erickson 2008; Miller et al. 2011) due to the often proclaimed potential risks associated with nonlocal genotypic material, namely outbreeding depression and adaptation (e.g., Kaye 2001; Mijangos et al. 2015). However, recent plant research suggests outbreeding depression and adaptation to be less common than formerly assumed (Edmands 2007; Leimu and Fischer 2008; Mijangos et al. 2015).

By using molecular tools to evaluate landscape restoration projects on compensatory sites several years after establishment we could demonstrate that the use of nonlocal seed provenances did not result in adverse population genetic effects on indigenous populations of *D. carota*. In the obligate outcrossing plant species we could detect negligible population differentiation between indigenous populations and populations restored with nonlocal seed material. No negative effects on allelic richness, selective sweeps, or reduced population genetic diversity could be observed, with one exception, where inbreeding effects are presumed. Even though no information about the geographic origin of the “nonlocal” seed material is available, we assume that for the common, outcrossing and generalist species the term “nonlocal” is a sweeping term which should not be narrowed down to geographic vicinity, as presently common in biodiversity conservation.

Decisive criteria for restoration projects are restoration objectives and goals, as well as the efficient use of resources considering costs and seed availability (Ehrenfeld 2000; Kaye 2001; Doede 2005; Wilkinson et al. 2008; Miller et al. 2011). On the basis of the population genetic analysis conducted here, there are no obvious objections against the current nature conservation practice to use the 10‐fold cheaper nonlocal seed material of *D. carota*. Moreover, this might also be the case for other common, generalist and outcrossing plant species.

## Data Archiving Statement

Data for this study are available at: to be completed after manuscript is accepted for publication.

## Conflict of Interest

None declared.
